# The Differential Early Responses of Human Leukocytes to Influenza Virus and Respiratory Syncytial Virus

**DOI:** 10.3390/pathogens14100974

**Published:** 2025-09-26

**Authors:** Norbert J. Roberts, M. Kerry O’Banion

**Affiliations:** 1 Division of Infectious Diseases and Immunology, Department of Medicine, New York University Grossman School of Medicine, New York, NY 10016, USA; 2 Division of Infectious Diseases, Department of Internal Medicine, University of Texas Medical Branch at Galveston, Galveston, TX 77555, USA; 3 Department of Neuroscience, University of Rochester School of Medicine, Rochester, NY 14642, USA; kerry_obanion@urmc.rochester.edu; 4 Department of Neurology, University of Rochester School of Medicine, Rochester, NY 14642, USA

**Keywords:** influenza virus, respiratory syncytial virus, human monocytes/macrophages, early immune responses

## Abstract

The current studies examined very early events associated with activation and initiation of a human immune response after sham exposure or exposure to influenza virus (IAV) versus respiratory syncytial virus (RSV), focusing on the function of a critical accessory cell for lymphocyte responses. Calcium mobilization by monocytes/macrophages was rapid and marked in response to exposure to IAV but was muted in response to RSV. Monocytes/macrophages exposed to IAV showed markedly enhanced expression of Cox-2 mRNA measured soon after exposure, whereas exposure to RSV resulted in reduced expression (relative to control cells). In contrast, expression of the constitutively expressed 2.8 kb Cox-1 mRNA was relatively constant. The 72/74 kDa/pl 7.5 protein doublet (product of the *Cox-2* gene) was identified in lysates of IAV-exposed monocytes/macrophages but not RSV-exposed monocytes/macrophages. The results demonstrate that human monocytes/macrophages show reduced responses to RSV, similar to previously demonstrated effects of RSV on lymphocyte responses. This relative lack of early responses may contribute substantially to the ability of RSV to re-infect individuals.

## 1. Introduction

Respiratory syncytial virus (RSV) is a major cause of respiratory infections worldwide, with the most severe cases occurring in very young and in elderly individuals [1,2,3,4]. The immune response to RSV does not prevent clinical re-infection and a single RSV isolate has been shown to cause repeated experimental symptomatic infections in adult volunteers [5]. It is thus important to delineate as much as possible the immune response to RSV to facilitate the development of effective preventive or therapeutic measures. Comparisons with the responses to influenza virus can be expected to support such investigations.

Both respiratory syncytial virus (RSV) and influenza A virus (IAV) can infect human peripheral blood mononuclear cells (PBMC), including both monocytes/macrophages and lymphocytes, during the immune response to viral challenge [6,7,8]. Our in vitro studies have examined virus-leukocyte interactions that might contribute to effective protection against re-infection with IAV and that might contribute to the ability of RSV to re-infect individuals who should be immune. Differential immune cell cluster formation (between monocytes/macrophages and lymphocytes) was noted as soon as an hour after exposure to IAV versus RSV [9], and this early observation was predictive of subsequent events and overall characteristics of the response to viral challenge [10,11]. For example, exposure to RSV resulted in reduced lymphocyte proliferation despite evidence of a virus-specific T lymphocyte frequency equivalent to that for IAV [11]. We undertook the current studies to examine very early events associated with activation and initiation of an immune response after exposure to the viruses, focusing on the function of monocytes/macrophages, critical accessory cells for lymphocyte responses. We studied calcium mobilization and Cox-2 expression as indicators of early responses of human monocytes/macrophages to influenza virus versus RSV, as well as the resulting effects on activation of lymphocytes when mixed PBMC were exposed sequentially to the two viruses.

## 2. Materials and Methods

### 2.1. Viruses

Previously described standard techniques were used to produce and measure RSV and IAV [12]. Influenza A/AA/Marton/43 (H1N1) virus was grown in embryonated hen’s eggs and plaque assayed in MDCK cells; RSV A subtype (Long strain, VR-26, ATCC, Manassas, VA, USA) was grown and plaque assayed in HEp-2 cells. Virus was quick-frozen for storage at −70 °C. Freshly isolated whole PBMC or monocytes/macrophages were exposed to RSV or IAV at a multiplicity of infection (MOI) of one for one hour in RPMI 1640, washed, and subsequently cultured in RPMI 1640 (Gibco, Invitrogen of Thermo Fisher Scientific, Waltham, MA, USA) with 10% defined FCS (Hyclone, Logan, UT, USA) at 37 °C. Previous studies determined that similar results would be observed with exposures at other MOI for both influenza virus and RSV [7,12].

### 2.2. Collection of PBMC and Purified Monocytes/Macrophages and Exposure of Cells to Virus

Informed consent for withdrawal of blood was obtained from all donors, and these studies were approved by the Institutional Review Boards of the University of Rochester and the University of Texas Medical Branch. Donors were healthy and ranged in age from 18 to 45 years of age with no recent (within 2 months) illnesses or use of medication or receipt of vaccines. Each experiment for a study used a different donor. All comparisons were performed with autologous cell preparations. It was expected that all donors experienced past in vivo exposure to RSV as well as IAV [11,12]. Furthermore, when tested, donors had equivalent evidence of a virus-specific T lymphocyte frequency to the two respiratory viruses [11]. PBMC were separated by Ficoll-Hypaque sedimentation and maintained in RPMI 1640 supplemented with 10% FCS and 1% pen/strep. Cells were counted and viability was determined (a) by the ability to exclude trypan blue under light microscopy or (b) by the use of propidium iodide with analysis by fluorescent microscopy and flow cytometry.

Purified monocytes and monocyte-derived macrophages were obtained by adherence of PBMC in plastic culture dishes, followed by extensive washing to remove nonadherent cells, and scraping and collection of adherent cells with a rubber policeman. In a subset of experiments, purified monocytes were obtained from PBMC by countercurrent centrifugal elutriation using previously described techniques with minor modifications [13,14]. Elutriation was conducted at 4 °C using a Beckman J2-21 centrifuge with a JE-6 elutriator rotor and Multiperplex pump with fine velocity control (LKB Instruments, Gaithersburg, MD, USA), with rotor and flow speeds based on the earlier studies. Monocytes were obtained from the “blow-out” fraction of elutriation (by turning off the rotor after elutriation of lymphocytes). There were no differences in results when comparing monocytes/macrophages obtained by adherence with those obtained by elutriation.

### 2.3. Flow Cytometry Analyses of Cell Phenotype and Activation Markers

Acquisition and analysis were performed using a FACScan flow cytometer and CellQuest software v3 (Becton Dickinson, Franklin Lakes, NJ, USA). The purity of cells used for the different assays for viral infection was always confirmed by direct immunofluorescent staining and two-color flow cytometry (FACScan, Becton Dickinson, Franklin Lakes, NJ, USA) analysis. Monoclonal antibodies identifying CD3+ T lymphocytes (anti-Leu-4) and CD14+ monocytes/macrophages (anti-Leu-M1), as well as isotype control antibodies (Becton Dickinson), were used in such analyses. Cell surface expression of CD69 was assayed using a monoclonal antibody conjugated to phycoerythrin (PE; Clone L78; Becton Dickinson). For each flow analysis, 10,000 total cells were examined.

### 2.4. Analysis of Calcium Mobilization by Monocytes/Macrophages

Monocytes/macrophages were loaded with fluo-3/AM (Molecular Probes, Eugene, OR, USA) and assayed using flow cytometry according to the manufacturer’s guidelines. F max was determined using ionomycin; F min was determined using Mn*2/Fluo-3.

### 2.5. Northern Blot and Giant 2-D Gel Analyses for Cox-2 mRNA and Protein

Total cellular RNA was obtained from sham-exposed and influenza virus (IAV)-exposed or RSV-exposed cells using described methods [15,16]. After prehybridization, Northern blots were hybridized with a ^32^P-labeled DNA strand complementary to *Cox-1* or *Cox-2*. Each blot was hybridized subsequently using a probe for the human cellular gene product beta-tubulin, derived from a cDNA cloned into the pBluescript II KS (+) vector (Stratagene, LaJolla, CA, USA), as a control for gel loading.

For gel analysis of the proteins, monocytes/macrophages were labeled in Dulbecco’s modified Eagle’s medium without methionine (GIHCO) plus 200 pCi/ mL Tran^35^S-label (>1000 Ci/mmol; ICN) for 15 or 30 min. Incorporation of label into proteins was determined by trichloroacetic acid precipitation. Details of the giant two-dimensional gel electrophoresis system have been described previously [17]. The discovery and identification of Cox-2 in 2D gels has been described extensively [18,19], as well as the characteristic finding of the Cox-2 protein on the 2-D gels: a p72–74 (72–74 kDa) doublet (pI 7.5) [19].

## 3. Results

### 3.1. Calcium Mobilization by Sham-, Influenza-, and RSV-Exposed Monocytes/Macrophages

**Figure 1:**
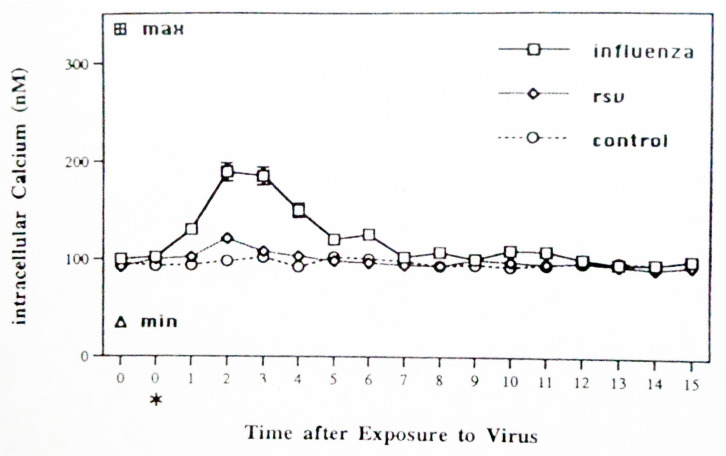
Calcium mobilization by sham-, influenza-, and RSV-exposed monocytes/macrophages. Elutriation-purified cells were sham-exposed (control) or exposed to the virus at the time indicated (*) and assessed for calcium mobilization at the times indicated (minutes) using flow cytometry with fluo-3. Mean results +/− SD are shown for four experiments. Data from 10,000 gated cells were collected for each cell preparation.

The first series of experiments examined calcium mobilization by sham-, influenza-, and RSV-exposed monocytes/macrophages. Elutriation-purified cells were sham-exposed (control) or exposed to the virus at the time indicated (*) in Figure 1 and assessed for calcium mobilization at the times indicated (in minutes) using flow cytometry with fluo-3. The data showed rapid mobilization of intracellular calcium by the monocytes/macrophages exposed to IAV whereas RSV-exposed cells showed a very small and much more transient response.

### 3.2. Cox-2 mRNA and Protein Expression by Virus-Exposed Monocytes/Macrophages

**Figure 2:**
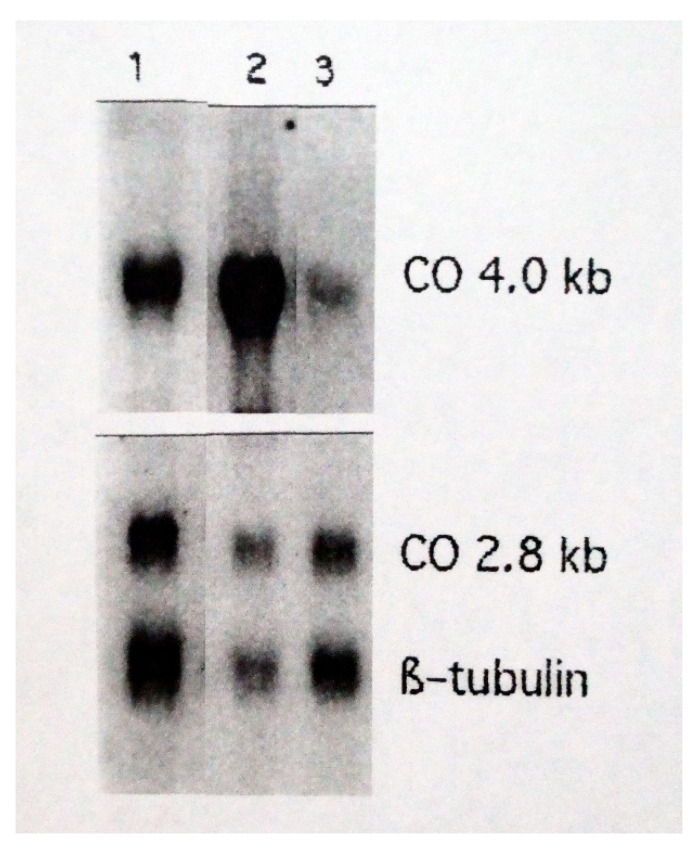
Cox-2 mRNA expression by sham-exposed or influenza- or RSV-exposed monocytes/macrophages. Cells were collected 4 h after sham exposure (Lane 1) or exposure to IAV (Lane 2) or RSV (Lane 3) and cellular mRNA was probed in Northern blots for expression of inducible Cox-2 (4.0 kb CO) and constitutively produced Cox-1 (2.8 kb CO) and beta-tubulin.

In a second series of experiments examining early effects of RSV on monocyte/macrophage function, we assessed Cox-2 mRNA expression by sham-exposed or influenza- or RSV-exposed monocytes/macrophages. We examined the virus-induced expression of this transcriptionally regulated Cox-2 gene. Cells were collected 4 h after sham exposure (lane 1) or exposure to IAV (lane 2) or RSV (lane 3) and cellular mRNA was probed in Northern blots for expression of inducible Cox-2 (4.0 kb CO) and constitutively produced Cox-1 (2.8 kb CO) and beta-tubulin (Figure 2). The data showed rapid, enhanced expression of Cox-2 mRNA by cells exposed to IAV, whereas exposure to RSV resulted in reduced expression of Cox-2 relative to sham-exposed cells.

**Figure 3:**
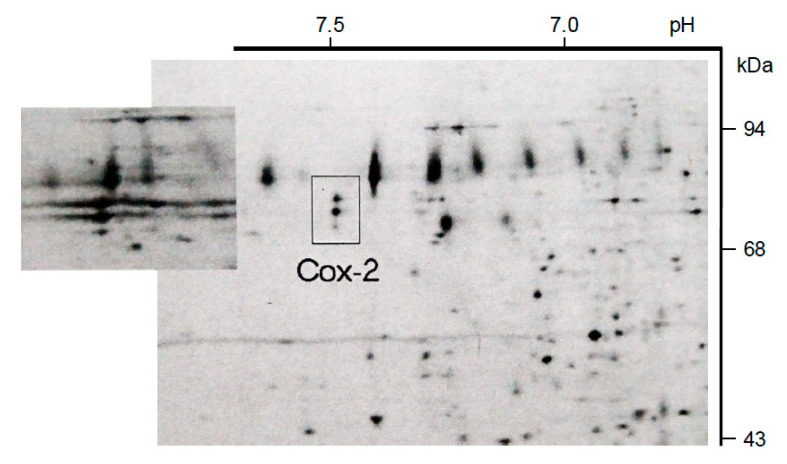
Cox-2 protein expression by IAV-exposed monocytes/macrophages. Cell lysates (0.5 h pulse) were collected 1.5 h and 4.5 h (data shown) after sham exposure or exposure to IAV (Figure 3) or RSV. Lysates were analyzed using giant 2D gel electrophoresis, and the 72/74 kDa/pl 7.5 protein doublet (product of Cox-2) was identified in lysates of IAV-exposed cells 1.5 h (inset figure) and 4.5 h (indicated in figure) after exposure but was not detected in the giant gels of lysates of RSV-exposed cells at either time after exposure.

We also examined Cox-2 protein expression by the monocytes/macrophages. Cell lysates (0.5 h pulse) were collected 1 h and 4 h after exposure and were analyzed using giant 2D gel electrophoresis. The 72/74 kDa/pI 7.5 protein doublet (product of *Cox-2*) was identified in lysates of influenza-exposed cells collected at both 1 h and 4 h (Figure 3) but was not detected in lysates of RSV-exposed cells at either time after exposure.

### 3.3. Sequential Exposure of PBMC to RSV and IAV and Analyses of Lymphocyte Activation

**Figure 4:**
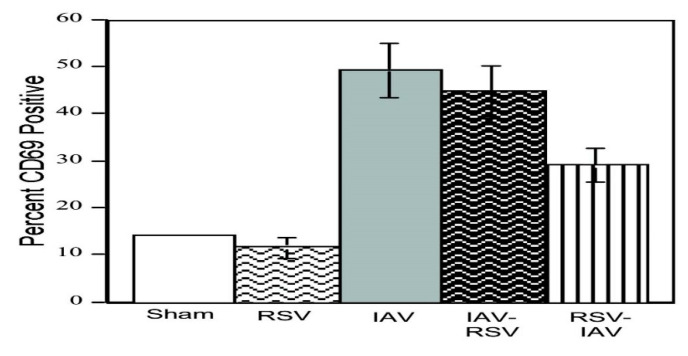
PBMC from adult volunteers were exposed to influenza (IAV) or RSV for one hour and then exposed for one hour to the alternate virus (IAV-RSV and RSV-IAV, respectively). Cells were then fixed and stained for CD3 phenotyping and CD69 expression at 24 h. Flow cytometric analysis was performed to determine the percentage of CD3+ lymphocytes that positively expressed CD69. Data from 10,000 gated cells were collected for each cell preparation. Mean results +/− SD are shown for two separate experiments.

It is reasonable to be concerned with whether an early immunosuppressive response to RSV might adversely affect responses to a second challenge for the host. In preliminary experiments to examine such a possibility, unseparated PBMC, containing both monocytes/macrophages and lymphocytes, were exposed to both RSV and IAV sequentially for one hour with no interval between the exposures. CD3+ lymphocyte expression of the early CD69 activation marker was measured (Figure 4).

## 4. Discussion

It is important to examine and understand the activation and initiation of a human immune response after sham exposure or exposure to IAV versus RSV. Previously published evidence indicated an early PBMC commitment to an immunosuppressed state in response to RSV versus activation and proliferation in response to IAV, notably measuring the impact on lymphocyte responses [10,11]. Additional studies have been warranted to examine effects of the viruses on key accessary cells for such lymphocyte responses. The current studies examined calcium mobilization and Cox-2 expression as indicators of early responses of monocytes/macrophages to IAV versus RSV, as well as the resulting effects on activation of lymphocytes when unseparated PBMC were exposed sequentially to the two viruses. The results indicated that there are very early differences in responses of human monocytes/macrophages to the two viruses.

Calcium mobilization is one of the earliest responses of monocytes/macrophages to challenge and, therefore, an appropriate indicator of the rapid differentiation of response to IAV versus RSV. Calcium mobilization controls diverse cellular processes, including proliferation and differentiation, transcription, cellular metabolism, and cell death [20,21]. Earlier studies using murine fibroblasts showed that a calcium ionophore stimulates synthesis of prostaglandin G/H synthase (PGHS, cyclooxygenase) protein and the 4-kh mRNA termed *Cox-2* [22]. Experiments showed a greater than 10-fold increase in PGHS protein synthesis when cells were treated with the calcium ionophore A23187.

PGHS is the first enzyme in the conversion of arachidonate to prostaglandins and plays a key role in immune and inflammatory responses [22,23]. Two pools of cyclooxygenase exist: a constitutively expressed pool (termed Cox-1) and a hormone-sensitive inducible pool (Cox-2). Cox-2 is a very early monocyte/macrophage response gene [22,24]. Its expression could have significant effects on cellular commitment to a proliferative versus an antiproliferative response to challenge. Prostaglandins have been recognized to have pleiotropic effects on immunological responses, clearly not limited to suppressive activities [23,25]. Thus, for example, anti-PGE antibodies have been shown to inhibit development of cell-mediated immune responses, whereas other studies have shown that prostaglandins inhibit lymphocyte proliferative responses as well as the production of cytokines [23].

CD69 expression by sham-, IAV-, and RSV-exposed CD3+ T lymphocytes exposed as PBMC was examined in experiments with sequential exposure of PBMC to the two viruses. The CD69 activation marker is one of the earliest antigens to appear upon activation of PBMC, appearing within two hours after stimulation [26,27,28]. The CD69 antigen of human lymphocytes is a calcium-dependent carbohydrate-binding protein [27]. Some reports have shown increases in the percent of cells expressing CD69 as early as 0.5 h after stimulation [29,30]. The results suggested that the very early differential responses of the accessory monocytes/macrophages to IAV versus RSV are reflected in turn by the differential lymphocyte activation responses. Once the cells were activated in response to IAV, there was little effect on the early activation responses of lymphocytes in the PBMC by immediately subsequent exposure to RSV. In contrast, exposure to RSV resulted in reduced activation of the lymphocytes when the PBMC were exposed immediately subsequently to IAV. Thus, the differential immune responses to the two viruses were determined very early after exposure.

Murine models have been used to demonstrate the rapid and substantial recruitment of peripheral blood mononuclear cells, both monocytes/macrophages and lymphocytes, to the lung after a respiratory virus challenge [31,32,33,34]. These recruited cells play important roles in defense against, and recovery from, the virus infection [32,34]. Tissue macrophages can be heterogeneous and differ in biological functions [35]. In several series of published studies, human alveolar macrophages were compared to autologous peripheral blood-derived monocytes/macrophages for immune functions including support of lymphocyte responses. The alveolar macrophages were shown to be less effective at supporting lymphocytes responses to mitogen or to antigens, including influenza virus antigens. This finding appeared to be due to active suppression by the alveolar macrophages rather than a deficiency in accessory cell function [36]. The alveolar macrophages produced very little or no IFN in response to infectious influenza virus in experiments in which peripheral blood-derived monocytes/macrophages produced substantial titers of IFN [36]. The latter finding may be due to a characteristic of the alveolar macrophages that has been reported more recently [37], namely the observation that human alveolar macrophages may not be susceptible to direct infection by a human influenza virus. The cells can be infected indirectly in the setting of exposure in the presence of antibody that binds the challenging strain of virus. Thus, there are major differences between the functions of alveolar macrophages and peripheral blood-derived monocytes/macrophages, and the current studies should be considered more reflective of the latter cells that would be expected to be recruited to the respiratory tract in response to virus challenge.

Numerous studies have suggested that soluble monocyte/macrophage-derived factors other than prostaglandins may influence the immune response to RSV compared to influenza virus. An altered regulatory cytokine profile has been reported in cases of pediatric RSV infection [38] and the contribution of cytokines to tissue damage during RSV infection has been reviewed recently [39]. We have shown previously that RSV induces less interferon (IFN) production by human monocytes/macrophages than does influenza virus, and is also less sensitive to IFN activity compared to influenza virus [11,40]. In those experiments, influenza virus induced high titers of total IFN bioactivity, tran-scription of the IFN-α1 and IFN-β gene products, and production of IFN. In contrast, RSV induced minimal or no detectable total IFN activity, and the absence of IFN bioac-tivity could not be attributed to inhibitors of IFN activity. There was no detectable transcription of IFN-α or IFN-β gene products by the cells exposed to RSV. RSV-exposed MNL did produce small amounts of IFN-γ, consistent with prior sensiti-zation of the cell donors to the virus, but titers were substantially lower than those in-duced by influenza virus [11,40]. In contrast to the reduced production of IFNs in re-sponse to RSV compared to influenza virus, previous studies have shown that RSV-exposed monocytes/macrophages produce greater amounts of IL-6 and TNF-α than cells exposed to influenza virus [41]. In addition, although both RSV- and influ-enza virus-exposed monocytes/macrophages produce IL-1 after exposure, RSV induces a greater expression of IL-1 inhibitors, resulting in a net reduction in support of lym-phocyte responses to the virus [10]. All these potentially inhibitory factors may con-tribute to reduced protection of the host from re-infection by RSV.

## 5. Conclusions

The current studies indicate that human immune responses to challenge by IAV and RSV differ substantially very early after exposure, affecting the function of monocytes/macrophages as well as lymphocytes. The results demonstrate that human monocytes/macrophages show reduced responses to RSV, potentially leading to the previously demonstrated effects of RSV on lymphocyte responses when the cells are exposed as unseparated PBMC. The cumulative effects on both monocytes/macrophages and lymphocytes may contribute substantially to the ability of RSV to re-infect individuals who should be immune to the challenge.

## Data Availability

The original contributions presented in this study are included in the article. Further inquiries can be directed to the corresponding author.
